# S13-3: ”I’m just one of the runners, one of the walkers”. Exploring the experiences of participants who are vision impaired at parkrun in Ireland

**DOI:** 10.1093/eurpub/ckae114.257

**Published:** 2024-09-26

**Authors:** Joan Ryan

**Affiliations:** Technological University Dublin, Dublin, Ireland

## Abstract

**Purpose:**

The benefits of physical activity are well established but people who are vision impaired are known to have low levels of participation. This qualitative research explores the experiences of nine people who are vision impaired at parkrun, a free, weekly 5km, mainstream community running and walking event.

**Method:**

Semi structured interviews were conducted with nine parkrun participants who were vision impaired who had each participated in a minimum of 10 events. The results were analysed using the thematic analysis process of Braun and Clarke.

**Results:**

Three main themes were identified from the data. The participants felt they were welcomed, in terms which they felt comfortable with. They felt that their wellbeing and health were enhanced by participation. They felt a sense of belonging within a mainstream community. Their successful participation enabled them to feel the benefits of physical activity from not just a biomechanical functioning perspective, but they also felt benefits to their psychosocial wellbeing which reached beyond the 5km weekly event.

**Conclusion:**

This data gives valuable insight into the perspective of people with disability within a mainstream physical activity event. It contributes to new insights on how inclusion is defined and achieved using the voice of people with vision impairment. It also provides an important contribution to the growing body of academic research on parkrun.

**Funding:**

The author is supported by a fees only PhD scholarship funded by Topcon and Vision Sports Ireland. This research is conducted as part of a PhD studentship at TU Dublin.

